# Feature-based attentional weighting and spreading in visual working memory

**DOI:** 10.1038/srep42384

**Published:** 2017-02-24

**Authors:** Marcel Niklaus, Anna C. Nobre, Freek van Ede

**Affiliations:** 1 Department of Psychology, University of Zurich, Binzmühlestrasse 14, 8050 Zurich, Switzerland; 2 Department of Experimental Psychology, University of Oxford, Tinbergen Building, 9 South Parks Road, Oxford OX1 3UD, UK; 3 Oxford Centre for Human Brain Activity, Department of Psychiatry, Warneford Hospial, Oxford OX3 7JX, UK

## Abstract

Attention can be directed at features and feature dimensions to facilitate perception. Here, we investigated whether feature-based-attention (FBA) can also dynamically weight feature-specific representations within multi-feature objects held in visual working memory (VWM). Across three experiments, participants retained coloured arrows in working memory and, during the delay, were cued to either the colour or the orientation dimension. We show that directing attention towards a feature dimension (1) improves the performance in the cued feature dimension at the expense of the uncued dimension, (2) is more efficient if directed to the same rather than to different dimensions for different objects, and (3) at least for colour, automatically spreads to the colour representation of non-attended objects in VWM. We conclude that FBA also continues to operate on VWM representations (with similar principles that govern FBA in the perceptual domain) and challenge the classical view that VWM representations are stored solely as integrated objects.

Attention pertains to the fundamental cognitive process of prioritizing relevant over irrelevant information and is, as such, critical for adaptive behavior^1^. Whereas attention has been studied in its many forms for many decades in the perceptual domain, it has more recently become clear that attention also continues to operate on mental representations held in visual working memory (VWM)^2^,^3^. Retro-cue studies have directed attention to select a particular object in VWM, either by directing attention to a spatial location^2^ or by using non-spatial retro-cues such as an object’s colour^4^, shape^5^ or category^6^. Here, we investigated whether another form of attention also continuous to operate during VWM, namely: feature-based attention (FBA), which describes the deployment of attention to relevant feature values (e.g. blue) or feature dimensions (e.g. colour) within objects, and independent of spatial location^7^. Specifically, we address whether FBA retro-cues can up- and down-regulate relevant and irrelevant features within multi-feature objects in VWM.

Modulation of performance in VWM tasks by FBA would be incompatible with the notion that integrated objects serve as the unit of VWM representations^8^,^9^. This standard account has already been challenged by the finding that errors in the recall of different features of objects in VWM can be independent; even when one feature of a multi-feature object is shown to be forgotten, another feature may still be recalled^10^,^11^. Furthermore, ecological arguments can be proposed for attention to continue to operate in VWM at the feature level, since keeping multiple features per object is costly^12^,^13^,^14^. We hypothesize, therefore, that FBA is capable of dynamically weighting feature representations within objects held in VWM.

In addition to attention to specific feature attributes, it has become clear that FBA can also be directed at dimensions^15^,^16^,^17^. The potential relevance of a feature-dimension level has recently also been postulated for VWM, in the form of a dimensional feature-bundle model (DFB)^18^, which contains a layer that represents entire feature dimensions in addition to lower level specific feature stores whose attributes are bound at the higher object-level^19^. Based on these observations, we hypothesize FBA may not only affect WM representations by facilitating individual feature representations, but perhaps also by weighting entire feature dimensions – possibly by up-regulating neuronal excitability in brain areas processing a particular feature dimension^20^,^21^,^22^,^23^. A prediction that follows from this work is that the hypothesized biasing effect of FBA in VWM will be stronger if, for different mnemonic objects, attention is directed to features within the same dimension, in comparison to across different dimensions.

An interesting characteristic of FBA in the perceptual realm is its global nature. That is, processing of an attended feature attribute^24^,^25^ or dimension^26^,^27^,^28^ is enhanced throughout the visual field, independent of the spatial focus of attention. Based on this work, we finally hypothesized that the influence of FBA in VWM may similarly be a global influence that will automatically spread to other (uncued) objects, with regard to their feature representations in the same dimension.

We thus set out to investigate three key hypotheses. First, features within multi-feature objects can be flexibly up- and down-regulated based on current relevance. Second, FBA in VWM is most efficient if directed within the same dimension for different objects. Third, feature dimension weighting spreads automatically to non-attended objects in VWM. To this end, we employed a series of VWM tasks that deployed FBA retro-cues and required a continuous reproduction of the colour or orientation of probed objects.

## Results

### Feature-dimension-based retro-cues improve representations in the more relevant feature dimension, at the expense of the less relevant feature dimension

**Figure 1:**
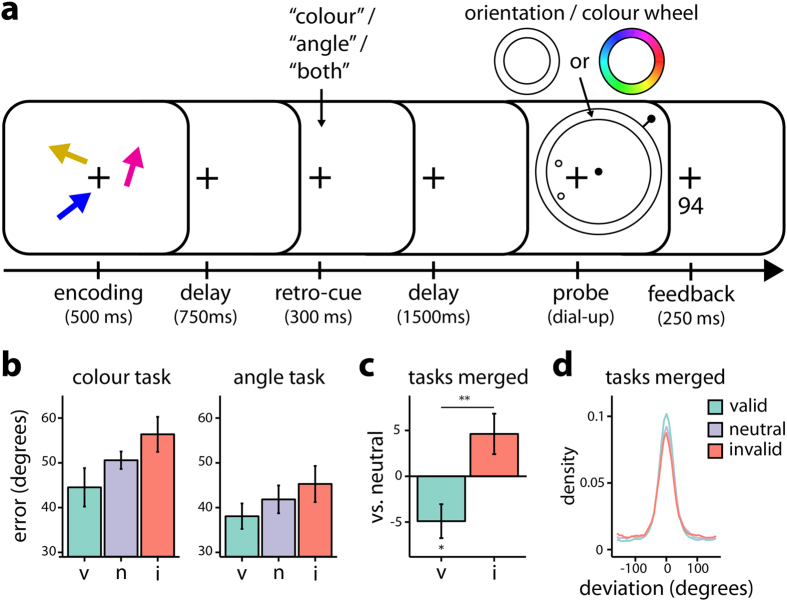
Procedure and results Experiment 1. (**a**) Experimental procedure. After encoding three coloured arrows, either an informative retro-cue (the word “colour” or “angle”) was presented that indicated with 75% validity which feature dimension was going to be probed, or a neutral retro-cue (the word “both”) was presented that indicated that either feature dimension was equally likely to be probed. Retro-cues were informative for all objects, and individual objects remained equally likely to be probed. Participants reported the dimension indicated by the nature of the wheel for the object indicated by the filled circle (right upper object in schematic), followed by performance feedback on a scale from 0 (bad) to 100 (perfect). (**b**) Mean errors for valid (v), neutral (n) and invalid (i) cues for the colour and orientation dimensions (N = 20). Errors bars depict 95% within-subjects confidence intervals^54^. (**c**) Data were normalized by subtracting the average value in the neutral condition from the valid and invalid conditions before averaging these data between the colour and the orientation tasks. Asterisks indicate significant results of a one-sample *t*-test of normalized benefits (v, valid - neutral) and costs (i, invalid – neutral) against zero, *p < 0.05, **p < 0.01, ***p < 0.001. Error bars depict ±1 standard error of the mean (SEM). (**d**) Density plot of response deviations relative to the probe’s true feature value, averaged across dimensions, for the different cueing conditions.

In Experiment 1, participants held three coloured arrows in memory and were probed to reproduce either the colour or orientation of one of them after a delay on a continuous wheel (Fig. 1a). A filled black circle indicated the probed item and the nature of the wheel determined the dimension that had to be reported: a blank and a coloured wheel prompted an orientation and a colour recall, respectively. Participants responded using a computer mouse that controlled the wheel’s handle. We measured the absolute error between the true and the reported value in degrees. While the objects were held in VWM, either an informative retro-cue was presented, which indicated the feature dimension that was most likely going to be probed (without informing which object would most likely be probed), or a neutral retro-cue was presented. We expected to find better working memory performance when the dimension was validly cued, in comparison to when the dimensional cue was neutral or invalid. To assess this hypothesis, the means of participants’ reproduction errors (absolute deviation from the target colour/orientation in degrees) were submitted to a 3 (cue validity: valid, neutral, invalid) × 2 (dimension: colour, orientation) repeated-measures analysis of variance (ANOVA).

Figure 1b depicts the mean errors in degrees for valid, neutral and invalid cues, separately for the colour and orientation dimensions, and suggests an inverse relationship between errors and the probability of being probed in a certain dimension. The analysis of errors yielded a main effect of cue validity [*F*(1.6, 29.6) = 8.11, *p* = 0.003, 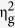 = 0.039]. Post-hoc contrasts revealed lower mean errors for valid than for invalid cues [*t*(38) = −4.03, *p*_hsd_ < 0.001], whereas the comparisons between valid vs. neutral cues [*t*(38) = −2.07, *p*_hsd_ = 0.109] and neutral vs. invalid cues [*t*(38) − 1.95, *p*_hsd_ = 0.138] were not significant. Importantly, however, in line with the claim that valid and invalid cues are associated with a benefit and cost in working memory performance, respectively, the linear contrast for the effect of validity was also significant [*t*(38) = 4.03, *p* < 0.001]. This was further confirmed by an alternative approach to investigating the benefits and costs of dimensional cues, as depicted in Fig. 1c. For this analysis, we calculated a single normalized benefit as the difference between the mean error in valid and neutral cues per dimension, and then averaging across the two dimensions. Similarly, a single normalized cost was defined as the difference in errors between invalid and neutral cues. One sample *t*-tests against zero yielded a significant benefit [*t*(19) = −2.64, *p* = 0.016], a nearly significant cost [*t*(19) = 2.09, *p* = 0.050], and a significant difference between benefit and cost, [paired *t*(19) = −3.27, *p* = 0.004].

We also compared performance on both dimensions and found better performance in reporting the orientation in contrast to the colour dimension [*F*(1, 19) = 8.51, *p* = 0.009, 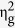 = 0.049]. Despite this difference, however, the effects of cue validity did not differ across the two dimensions, as indicated by a non-significant interaction between validity and dimension [*F*(1.9, 35.2) = 1.04, *p* = 0.359, 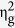 = 0.002].

The response-deviation density plots in Fig. 1d reveal that the uncued dimension was not dropped, but instead that the effects of FBA retro-cues in VWM operate in a subtle manner on the fidelity of representations. Taken together, these results indicate that FBA can enhance VWM representations in the more relevant feature dimension, and suggest that this occurs at the expense of the less relevant feature dimension.

### Retro-cueing benefits are larger when cued feature dimensions are shared between objects

**Figure 2:**
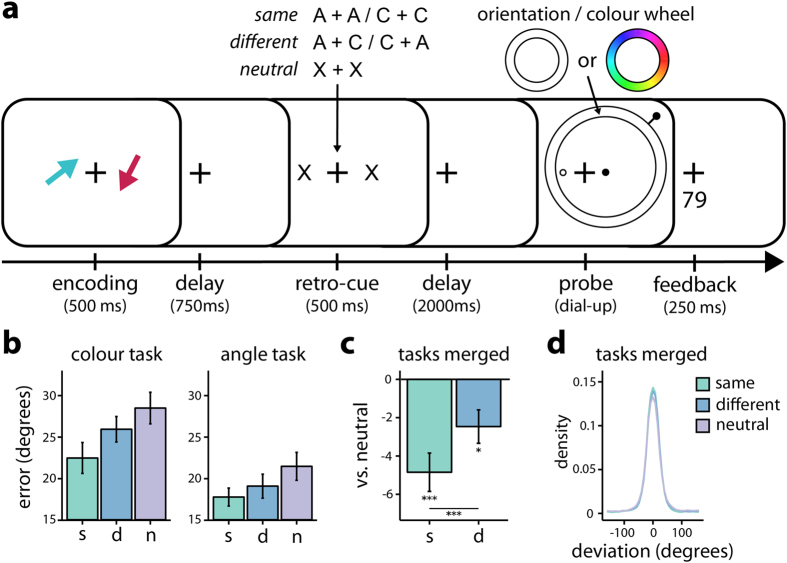
Procedure and results Experiment 2. (**a**) Experimental procedure. For each of the two encoded coloured arrows, either a neutral retro-cue (“X”, 33%) was presented, or a retro-cue that indicated which feature dimension (A = angle, C = colour) would be tested, if that object would be probed. In the same condition (33%), both objects were cued with regard to the same dimension, whereas in the different condition (33%), each object was cued with regard to a different dimension. Response procedure as in Experiment 1. (**b**) Mean errors for same (s), different (d) and neutral (n) cues for the colour and orientation dimensions (N = 20). (**c**) Normalized benefits for same (s, same – neutral) and different (d, different – neutral) cues. (**d**) Density plot of response deviations in all cueing conditions. Conventions as in Fig. 1.

It has been proposed that VWM access is easier when the to-be-remembered features of all objects are in the same dimension^18^. Based on this work, in Experiment 2 we investigated whether the same principle applies to the allocation of attention. To this end, we modified the cueing procedure of Experiment 1 as depicted in Fig. 2a. For each of the two presented memory stimuli, we separately presented a feature-dimension retro-cue that indicated with 100% certainty which feature dimension would be tested, if that object would be probed (50% chance per object). The critical manipulation here was that both objects would be cued either with regard to the same dimension, or with regard to different dimensions. Directing attention within the same dimension was expected to be associated with a larger retro-cueing benefit. To investigate this hypothesis, a 3 (cue type: same, different, neutral) × 2 (dimension: colour, orientation) repeated-measures ANOVA was performed on means of participants’ errors.

Figure 2b depicts the mean errors for all experimental conditions (Fig. 2d for the associated density plots). Relative to neutral retro-cues, both same and different dimension retro-cues benefited performance, with this benefit being strongest when the cued feature dimensions are shared between the two objects. This is supported by a significant main effect of cue type [*F*(1.4, 27.4) = 17.25, *p* < 0.001, 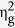 = 0.056], with post-hoc contrasts confirming a cueing benefit for same vs. neutral cues [*t*(38) = −5.87, *p*_hsd_ < 0.001], as well as for different vs. neutral cues [*t*(38) = −2.98, *p*_hsd_ = 0.013]. Crucially, same cues were additionally associated with a larger benefit than different cues [*t*(38) = −2.89, *p*_hsd_ = 0.017]. Figure 2c again depicts the normalized benefits relative to the neutral conditions and confirms a significant benefit for same [*t*(19) = −4.84, *p* < 0.001] as well as for different cues [*t*(19) = −2.82, *p* = 0.011], with a significant difference between them [paired *t*(19) = −4.50, *p* < 0.001]. Moreover, while the main effect of dimension [*F*(1, 19) = 20.96, *p* < 0.001, 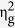 = 0.126] again revealed better performance on reporting the orientation than colour dimension, cue type and dimension did not interact with one another [*F*(2.0, 37.7) = 0.77, *p* = 0.470, 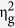 = 0.004], indicating that the effects of cue type did not differ across the two dimensions.

It is conceivable that participants required additional time to process and utilize the attentional cues when these were directed across two in comparison to within one dimension. If at time of probe presentation such processes are finished for same- but not for different-dimensional cues, then this may lead to additional interference and thereby worsen performance following different-dimensional cues. To investigate this possibility, we reasoned that this would result in delayed onset of the reproduction report for different dimension trials. Importantly, paired t-tests revealed that response-onset times for same (*M*_colour_ = 1.38 s, *M*_*orientation*_ = 1.17 s, *SD*_*colour*_ = 0.37, SD_orientation_ = 0.39) and different (*M*_colour_ = 1.33 s, *M*_*orientation*_ = 1.14 s, *SD*_*colour*_ = 0.36, SD_orientation_ = 0.37) cues did not differ, neither for the colour [paired *t*(19) = 1.38, *p* = 0.183] nor for the orientation dimension [paired *t*(19) = 0.67, *p* = 0.512].

Taken together, these results demonstrate that there is an additional benefit if attention can be directed to the same, as opposed to different feature dimensions for different objects in VWM.

### Feature-dimension-based attentional weighting of colour spreads to the colour of non-cued objects

**Figure 3:**
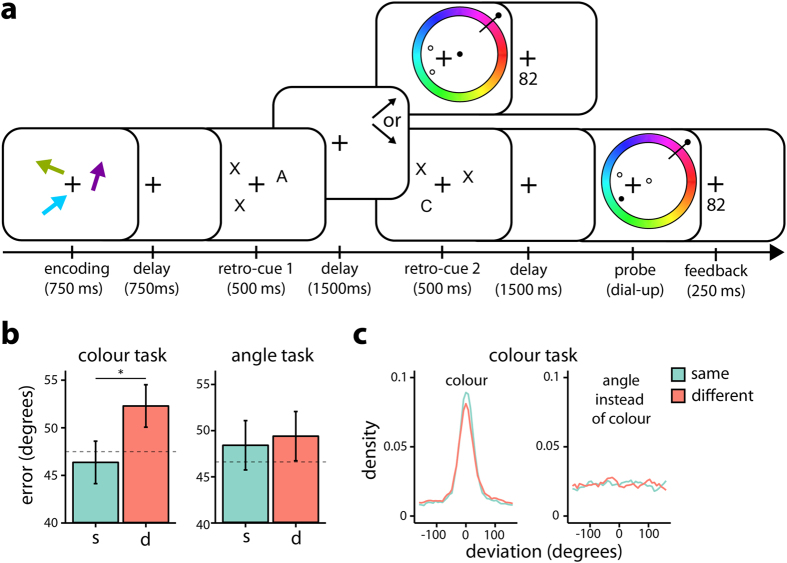
Procedure and results Experiment 3. (**a**) Experimental procedure. After encoding three coloured arrows, a retro-cue indicated which feature dimension (A = angle, C = colour) would be tested if the object would be probed “early”. Presentation of an “X” indicated that this object would not be probed immediately. In some trials, instead of probing “early”, a second retro-cue was presented that directed attention towards a feature of a previously uncued (“X”) object in “late” trials. The dimension cued with the second retro-cue could either be same as (50%) or different from (50%) the dimension cued with the first retro-cue. Participants were required to report the cued dimension for the object identified by the filled circle on a combined colour and orientation wheel. In half the trials, two (instead of one) “early” retro-cues were presented, but analyses collapsed across the number of early retro-cues (see Methods for details). (**b**) Mean errors in late trials for the colour and orientation dimension as a function of dimensional congruency between the second retro-cue and the first retro-cue (N = 20). The dashed black line shows the mean performance in early trials. (**c**) Density plot of response deviations in the colour dimension relative to the probed object’s true colour (left) or true orientation (right), for same and different trials. Conventions as in Fig. 1.

To investigate whether the global nature of FBA also extends to VWM, in Experiment 3 we asked participants to keep track of the cued feature associated with a specific object in order to report it on a combined colour and orientation wheel (Fig. 3a). Sometimes participants were probed on the initially cued feature (“early”), but other times a second retro-cue cued participants to switch their attention towards the same or different dimension of another object, in which case they were probed “late”. We focused on performance on late trials, and expected better performance when the second retro-cue was directed towards the same compared to a different feature dimension. A 2 (congruency: same, different) × 2 (dimension: colour, orientation) repeated measures ANOVA was performed on subject means of errors.

Figure 3b shows the mean reproduction errors for late trials when attention was redirected to a feature dimension that was the same or different to the dimension indicated by the first cue, separately for the colour and orientation dimension. In line with the notion of an automatic spread of FBA, the error analysis yielded a main effect of congruency (same/different) [*F*(1, 19) = 4.98, *p* = 0.038, 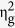 = 0.009] driven by better performance for same in comparison to different trials. Moreover, neither the main effect of dimension [*F*(1, 19) = 0.04, *p* = 0.838, 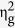  < 0.001] nor the interaction between dimensions and congruency [*F*(1, 19) = 1.95, *p* = 0.178, 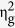 = 0.005] reached significance. However, despite the absence of a significant interaction between dimensions and congruency, it appears from Fig. 3b that the congruency effect is mostly driven by performance on the colour dimension. Indeed, dimension specific analyses yielded a congruency effect for the colour [*F*(1, 19) = 7.73, *p* = 0.012, 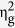 = 0.026] but not for the orientation dimension [*F*(1, 19) = 0.15, *p* = 0.702, 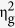 < 0.001].

Interestingly, for the colour dimension, we observed that performance in same trials was not statistically different from performance on early trials in which the colour dimension was probed [paired *t*(19) = 0.58, *p* = 0.563] (with the latter performance being indicated by the dashed line in Fig. 3b). In contrast, different trials showed worse performance [paired *t*(19) = 2.49, *p* = 0.022]. Thus, even though attention had to be re-directed to another object, if this refocusing of attention occurred within the same feature dimension, no significant cost was observed.

In order to verify our interpretation, it is important to rule out two additional possible sources that may contribute to the observed pattern in behaviour. First, because both dimensions were required to be reported on the same wheel, erroneously reporting the wrong dimension may occur. If so, it is feasible that this may occur more frequently in different trials in which the to-be-reported dimension changes with the second retro-cue. Focusing on the colour dimension data in which the effect was most pronounced, Fig. 3c depicts response deviations relative not only to the to-be-reported colour (left panel), but also to the not-to-be-reported orientation (right panel). Critically, these data reveal no evidence for erroneously reporting the object’s orientation in the colour task, nor for any difference between same and different trials with regard to such misreporting. Second, shifting to another dimension on different trials may take time, and if the probe would appear before such shifting was completed, increased interference may be expected by the probe onset. To address this concern, we again turned to our response-onset time data. Critically, paired t-tests indicated that response-onset times in the colour dimension did not differ between same (*M*_colour_ = 0.61 s, *SD*_colour_ = 0.21) and different (*M*_colour_ = 0.59 s, *SD*_colour_ = 0.21) trials [paired *t*(19) = 1.12, *p* = 0.276].

These results indicate that if attention is directed towards the colour of one specific object in VWM, this automatically facilitates the colour representations of the other objects in VWM – in line with a spread of FBA at the dimensional level.

## General Discussion

Over the past decade, retro-cueing has become a powerful paradigm for studying the influence of attention over internal representations held in VWM^3^. To date, however, this work has concentrated predominantly on retro-cues that allow for the selection of one out of several mnemonic representations (whose features are typically thought to be part of an integrated object-based representation). In a series of visual working memory tasks, we investigated the effects of feature-based retro-cues that cued either the colour or the orientation dimensions across multi-feature objects held in VWM. We show that FBA also continuous to operate on VWM representations, by improving VWM representations in the cued feature dimension, at the expense of the uncued dimension. Moreover, we demonstrate that attentional allocation is most efficient if, for different objects, FBA is directed within the same dimension in comparison to across different dimensions. Finally, we found that, at least for the colour dimension, attentional weighting operates in a global manner, spreading automatically to the colour representation of non-attended objects maintained in VWM. These findings parallel global FBA modulations observed in perception^24^,^25^,^26^,^27^,^28^. Because these data suggest that features can be independently prioritized to enhance performance, they also challenge the dominant view that the unit of VWM representation consists solely of integrated objects.

### Features and feature-dimensions of VWM representations

Based on the observation that VWM capacity for single feature objects was similar to memory capacity for multi-feature objects, VWM was proposed to operate on integrated object representations^8^,^9^. Recent studies, however, have started to challenge this view by demonstrating that increasing the number of features per object is costly^12^,^13^ and proposing that feature dimensions of multi-feature VWM objects may be stored with some degree of independence^29^ while being bound together by attention^30^. Consistent with the proposition of independent storage buffers, errors in reporting the colour and orientation of an object in VWM can arise largely independently in each feature dimension^10^,^11^. Complementing this work on forgetting, we here show attention can also be voluntarily deployed to facilitate performance for relevant feature-dimensions in order to best serve goal-directed behaviour.

Although we did not systematically manipulate working memory load, our results also have bearings on the debate regarding whether VWM capacity is governed by slots or a flexible resource^31^,^32^,^33^. Clearly, a very strict slot model, in which the unit of each slot is an integrated object, cannot account for our results. Indeed, our results may more readily be explained by resource models^34^,^35^ in which resources can be flexibly allocated to the attended at the expense of the unattended feature dimension. Still, we cannot exclude less stringent forms of slot models that incorporate some type of resource and/or feature-specificity within the slots^36^,^37^.

Our findings converge with dimensional effects observed in perception^16^ as well as VWM^18^,^38^. However, it should be noted that such dimensional effects do not necessarily justify a “representational” dimension-layer^18^, as conceptually no extra representational information is provided. Indeed, any object like *a green car* can be fully characterized by its object- and feature level attributes. However, as we will argue in the next section, the proposition of independent stores for feature dimensions may still be well suited for explaining the dimensional effects that we and others^11^,^18^,^39^,^40^ observed, especially when considering the potential neuronal mechanisms behind FBA in VWM.

### Potential neuronal basis of dimensional effects

Both Fougnie and Alvarez^11^ as well as Bays^41^ postulated that the independence they observed for performance on different features of multi-feature objects in VWM may reflect the fact that the feature level representations of a given object are supported by distinct neural populations. Accordingly, feature-specific errors may be attributable to stochastic noise at the level of feature-specific neuronal stores^11^. It is conceivable that our retro-cues were particularly helpful for overcoming this ‘noise’ within those neuronal populations tuned to the relevant feature dimension. In line with this speculation, attention has been shown to decrease noise correlations in populations coding for relevant stimuli^42^,^43^, as well as stimulus features^44^.

To accommodate for the dimensional effects observed in the current experiments, it is interesting to consider that FBA in the perceptual domain has been shown to modulate neuronal population that are specialized for processing a particular feature dimension, such as visual area V4 for colour and V5/MT for motion^20^,^21^,^27^,^28^. Moreover, the same targeted sensory areas support VWM^22^,^23^,^45^. Although not directly tested in the current experiment, such modulations provide a plausible neural basis for two particular effects we observed in the current study: (1) the enhanced cueing benefit when objects were cued in the same feature dimension in Experiment 2, and (2) the automatic spread of attention to the cued feature dimension (ic. colour) of non-attended objects in Experiment 3. FBA directed at only one population may be more efficient than directing FBA across two different populations, and up-regulation of the processing efficiency of the entire population may lead to the automatic spread of attentional biasing to non-attended objects, within the feature dimension that is coded for by that neuronal population. Finally, we speculate that this neural account may also explain why we observed a much more prominent spreading of attention for colour, as compared to orientation information in Experiment 3. Colour involves a higher-level visual attribute that is likely to have a more dedicated neuronal population, such as area V4. Orientation, in comparison, is a visual attribute that is much more tightly linked with retinotopic coordinates, and it is not evident that any particular visual area beyond the primary visual cortex is dedicated for processing this feature dimension. To further test these speculative ideas, in future work, it would be interesting also to investigate the automatic spreading of feature-dimension-based attention to non-attended objects for other feature dimensions such as motion, contrast, and shape^11^.

### Relation to previous FBA studies in VWM

Whereas ample evidence exists for retro-cueing particular objects in VWM, previous evidence for an effect of dimension-based retro-cues has been scarce and ambiguous. In one recently published study by Pilling and Barrett^46^, retro-cueing a feature dimension yielded no effect on performance in a change-detection task, whereas retro-cueing-benefits were observed for a sameness detection. In the present task, we used a continuous reproduction task and demonstrate that the influence of feature dimension based retro-cues in VWM is robust, for both colour and orientation dimensions. Moreover, in parallel to our study, a recently published study with a similar task to our first experiment also reported that FBA can affect VWM performance, showing an increased rate of recalling the target item for valid in comparison to neutral dimension based retro-cues^38^. Here, we further show that such a FBA benefit comes with a cost for the non-cued dimension, that it may operate in a global manner, and that it is larger when the cued feature-dimension is shared between objects. Interestingly, in yet another related study, it was shown that attention to particular feature dimensions during a working memory task (in this case, by retrieving particular dimensions), may also impact the consolidation of information into long-term memory^47^. This suggests that the FBA effects on VWM that we observed here during VWM delay, may not only have a facilitatory influence in the short term, but may even carry-over to long-term memory benefits of relevant features. Future work is required to test this exciting possibility.

### Sources of errors

Following up on our demonstration that FBA can influence VWM performance, a logical next step will be to investigate the sources of errors contributing to these effects. Error distributions can be modeled as a mixture of three components^48^, which allows the estimation of the proportion of trials in which participants (a) recalled the probed item, (b) recalled a non-probed item from the memory list, or (c) guessed. Moreover, the model allows the estimation of the precision with which memory items are held. Due to the restricted numbers of trials per condition we were cautious about performing such analyses given the possibility of obtaining unreliable estimates of the relevant factors. Future research should aim to pursue this question by using a simpler design that allows for more trials per condition.

## Conclusion

We have shown that FBA continues to operate on VWM representations. In line with our three key hypotheses, we demonstrated that directing attention to feature dimensions of multi-feature VWM objects is associated with a benefit as well as a cost for relevant and irrelevant feature representations respectively. Moreover, in line with dimensional effects that are characterized best in the perceptual domain, we showed that FBA is most efficient if directed at the same dimension for all cued objects. Finally, in line with global influences of FBA in the perceptual domain^24^,^25^,^26^,^27^, we revealed that attentional weighting of the colour dimension automatically spreads to the colour of non-attended objects in VWM. A plausible explanation for these observations is that FBA operates at the level of the neuronal populations that specialize in the processing of relevant feature dimensions.

## Methods

### Participants

Across three experiments, we recruited three separate groups of healthy volunteers. Experiment 1 included twenty-one participants (11 females, age: *M* = 24, Range = 19–38). One participant was excluded because she reported closing her eyes during the working memory delay (thereby not processing the retro-cues). Experiment 2 included another pool of twenty participants (13 females, age: *M* = 22, Range = 18–29) and so did Experiment 3 (10 females, age: *M* = 25, Range = 18–39).

Experimental protocols received approval from the Central University Research Ethics Committee of Oxford. The experiment was conducted in accordance with their policy on research involving human participants and personal data. All participants provided informed consent, had normal or corrected-to-normal vision and reported normal colour vision. Participation was reimbursed £8 per hour. All three experiments each lasted 75 minutes and participants were debriefed after participation.

### Materials

The experiments were presented on a 23-inch monitor (1920 × 1080, 60 Hz) using the Psychophysics Toolbox^49^,^50^ in Matlab. Throughout the experiments, the background colour was set to grey (RGB = 0.78, 0.78, 0.78). Stimuli consisted of coloured arrows (length: 1.50°, width: 0.50° visual angle), at a viewing distance of 70 cm. For each trial, orientations were drawn randomly without replacement from 360 possible angles, and colours were drawn randomly without replacement from 360 colours created from the CIE L * a * b* colour model. The model’s luminance parameter was set to 70, a* and b* were set to 20 and 38 respectively.

### Experimental procedures

#### Experiment 1

Each trial (Fig. 1a for a schematic) began with the presentation of a central fixation cross for 500 ms, followed by the display of the study array containing three coloured arrows for 500 ms. The arrows were presented equidistantly on an invisible circle (diameter 4.91°) centered around fixation. The location of the three arrows varied randomly on a trial-to-trial basis. After a 750 ms delay, a retro-cue was presented for 300 ms centrally 2.35° above the fixation cross. In the cued condition either the word “colour” or “angle” was presented, and in the neutral condition the word “both” was displayed. Retro-cues cueing either feature dimension were valid in 75% of the cases, and only informed which dimension was most likely to be probed (i.e. in contrast to typical retro-cueing studies, cues never informed which object was most likely to be probed). Probes were presented 1500 ms after the retro-cue and contained two relevant pieces of information. First, at the locations where the arrows had been presented, three circles (diameter: 0.90°) were presented, of which one was filled. This filled circle indicated which object was probed. Second, a response wheel was presented centered on the probed location (diameter: 12.28°–14.53°, centered 2.46° away from screen center). The nature of the wheel indicated the dimension that had to be reported: a blank and a coloured wheel prompted an orientation and a colour recall, respectively. Participants responded using a computer mouse that controlled the wheel’s handle (length: 0.50°), whose initial position was randomly assigned. Response time was unlimited until participants moved the handle, which triggered the count-down of a 2500 ms dial up time, which was visualized by a sand clock (length and width: 1°). The position of the handle when participants clicked or when the time limit was reached was taken as the response. At the end of each trial, feedback on the performance in the trial (reproduction precision, re-scaled as a number between 0 and 100) was displayed for 250 ms. After a blank 500-ms inter-trial interval, the next trial started.

The experiment consisted of ten blocks, which were separated by self-paced breaks. A total of 432 trials were run after an initial completion of eight practice trials, which were discarded from analyses. The feature dimensions colour and orientation were probed an equal number of times. In a fifth of all trials, a neutral cue was displayed. For the remaining trials, the feature dimension was cued with 75% validity, yielding 144 validly cued, 48 invalidly cued, and 48 neutral trials for each of the two task dimensions. All trial types were randomly interleaved.

#### Experiment 2

In Experiment 2 (Fig. 2a for a trial-schematic), we always presented two, instead of three, coloured arrows, that were always presented on the left and right side of the fixation cross (distance from center: 2.46°). After a 750 ms delay, two retro-cues were simultaneously presented for 500 ms, one at each arrow’s location. The letter “C” or “A” was presented to indicate that the colour or the angle of this arrow would have to be reported (100% valid), if this arrow would be probed (50% chance for each arrow). In the same-dimension condition, the same letter was displayed for both objects (A-A and C-C). In the different-dimension condition, one object was cued colour and the other angle (A-C and C-A). In the neutral condition, two “X”s were displayed to indicate that all dimensions of the objects could be tested. The remaining sequence of events was the same as in Experiment 1.

The experiment consisted of ten blocks, which were separated by self-paced breaks. A total of 468 trials were run after an initial completion of ten practice trials, which were discarded from analyses. The feature dimensions colour and orientation were asked to be reported an equal number of times. Neutral, same-dimension and different-dimension retro-cues were presented an equal number of times and the feature dimension cue was 100% valid, yielding 78 trials per cue condition and dimension. All trial types were presented in random order.

#### Experiment 3

A schematic sequence of events on each trial is depicted in Fig. 3a. Until presentation of the first retro-cue, the procedure is identical to Experiment 1 (and so are all stimuli dimensions). The first retro cue was presented for 500 ms and presented either the letter “C” or “A” at a location to indicate that the colour or angle would be probed, if this object would be tested early. Items that would not be probed early were marked by “X” (as depicted in Fig. 3a). After a 1500 ms delay, either the cued object was probed on the cued dimension, or a second cue was presented at a previously uncued object’s location indicating which dimension and object were going to be tested instead, after an additional delay. This second retro-cue was presented for 500 ms and followed by a 1500 ms delay before the response wheel appeared. In contrast to Experiments 1 and 2, the response wheel was always coloured and thus only the cue indicated the dimension that had to be reported for the probed arrow, which was again identified by a filled dot appearing at the location of where the object had been presented. From the outset of a trial, all objects were equally likely to be probed. Accordingly, the chance of being probed initially was 33%, whereas the chance of receiving another retro-cue instead was 66%. The critical manipulation was that in those trials that received a second retro-cue (for a previously uncued object), either the same or a different dimension was cued (with same and different retro-cues being equally probable). Thus, while in these “late” trials participants were always required to shift their attention to another object, in half the trials they were additionally required to shift their attention to another feature-dimension, whereas in the other half of the trials they maintained their attention in the same feature-dimension.

In half the trials, two (instead of only a single) objects were retro-cued at the stage of the first retro-cue. In these trials, retro-cues always indicated the same dimension, and the chances of being probed initially or receiving another retro-cue reversed to 66% and 33%, respectively. To increase sensitivity, we collapsed trials across the number of early retro-cues. This was further justified by the lack of an interaction between the number of retro-cues presented early, and the congruency of the second retro-cue, with regard to the performance in the “late” trials of interest [*F*(1, 19) = 0.02, *p* = 0.895, 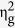 < 0.001].

The experiment consisted of ten blocks, which were separated by self-paced breaks. A total of 432 trials were run after an initial completion of 20 practice trials, which were discarded from analyses. The feature dimensions colour and orientation were asked to be reported an equal number of times. All trial types were randomly interleaved.

### General Data Analysis

For each trial we collected one main dependent variable: the angular response deviation in either colour or orientation space between the participant’s report and target object’s true feature value. For both dimensions, response deviations ranged between −180° and 180° and were converted into an absolute value, which we term “error”. For several control analysis, we also investigated response times, defined as the time between the onset of the probe screen and the start of the (time restricted) dial-up, which was triggered by movement of the mouse. All analyses were based on participant-specific condition averages. Trials with a response onset time above 4 s were discarded from analyses. Conditions were compared using an analysis of variance (ANOVA), as implemented in the afex package^51^ in R. Throughout the manuscript, degrees of freedom are Greenhouse-Geisser corrected in ANOVAs for repeated-measures factors with more than two levels. Furthermore, following Bakeman’s^52^ recommendations, we report 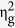 as an effect size measure for ANOVAs. Follow-up contrasts are obtained using the methods implemented in lsmeans^53^. For ANOVAs, these contrasts use Satterthwaite approximated degrees of freedom. To control for multiple testing, *p*-values of post-hoc contrasts are corrected using the Tukey’s HSD method and are denoted *p*_hsd_. The data and the analysis scripts for all experiments can be accessed in the Open Science Framework (https://osf.io/cz7xt/).

## Additional Information

**How to cite this article**: Niklaus, M. *et al*. Feature-based attentional weighting and spreading in visual working memory. *Sci. Rep.* **7**, 42384; doi: 10.1038/srep42384 (2017).

**Publisher's note:** Springer Nature remains neutral with regard to jurisdictional claims in published maps and institutional affiliations.
